# The clinical significance of CXCL16 in the treatment of advanced non‐small cell lung cancer

**DOI:** 10.1111/1759-7714.13387

**Published:** 2020-03-12

**Authors:** Yuji Shibata, Nobuaki Kobayashi, Takashi Sato, Kentaro Nakashima, Takeshi Kaneko

**Affiliations:** ^1^ Department of Pulmonology Yokohama City University Graduate School of Medicine Yokohama Japan; ^2^ Institute for Biomedical Sciences Shinshu University Kamiina Japan

**Keywords:** Bevacizumab, CXCL16, non‐small cell lung cancer, VEGF

## Abstract

**Background:**

Bevacizumab, a monoclonal antibody against vascular endothelial growth factor (VEGF)‐A, has shown efficacy in patients with advanced nonsquamous non‐small cell lung cancer (NSCLC). There are no identified or clinically validated biomarkers to determine the efficacy of bevacizumab. In this study, we assessed the adequacy of chemokine (C‐X‐C motif) ligand 16 (CXCL16) as a biomarker for patients treated with bevacizumab‐containing chemotherapy regimen.

**Methods:**

Patients diagnosed histologically with NSCLC were enrolled. Serial serum CXCL16 levels during treatment were measured by enzyme‐linked immunosorbent assay. The relationship between serum CXCL16 levels before and after treatment, progression‐free survival, and overall survival were analyzed. CXCL16 and VEGF‐A expressions in lung cancer tissue were also evaluated by immunohistochemical tests.

**Results:**

The median serum level of CXCL16 in these patients was 3.4 ng/mL, which was significantly higher than that in age‐matched healthy adults (2.2 ng/mL). Immunohistochemistry results showed that CXCL16 was predominantly localized in the tumor stroma, whereas VEGF was expressed in tumor cells. Including bevacizumab with chemotherapy led to lower CXCL16 levels post‐chemotherapy, which correlated with better response rates. In addition, evaluation of differences in serum CXCL16 levels before and after the first‐line chemotherapy showed that longer overall survival was achieved in patients who showed a larger decrease in serum CXCL16 levels.

**Conclusions:**

According to our findings, serum CXCL16 level was identified as a potential biomarker for the efficacy of therapy, including anti‐VEGF.

**Key points:**

Significant findings of the study

Patients with NSCLC whose serum CXCL16 levels decreased below 0.07 ng/mL after chemotherapy, showed longer overall survival than those without this decrease. Moreover, low CXCL16 levels corresponded to better response rates among patients with advanced NSCLC treated with bevacizumab‐containing chemotherapy.

What this study adds

Previously there were no identifiable predictive biomarkers to determine the efficacy of bevacizumab. Data from our findings identified serum CXCL16 level as a potential biomarker for the efficacy of bevacizumab‐containing chemotherapy.

## Introduction

Non‐small cell lung cancer (NSCLC) accounts for 85%–90% of all cases of lung cancer and patients with NSCLC are often diagnosed in the advanced stages.^1^ Bevacizumab, a monoclonal antibody against vascular endothelial growth factor (VEGF)‐A, has shown efficacy by inhibiting abnormal vascular growth in malignant tumors when added to the platinum‐doublet first‐line therapy for patients with advanced nonsquamous NSCLC.^2^ Although several studies have been conducted, none has identified or validated a clinically applicable predictive biomarker for bevacizumab efficacy for the treatment of NSCLC.^3^, ^4^, ^5^, ^6^, ^7^

The chemokine (C‐X‐C motif) ligand 16 (CXCL16) and its receptor, C‐X‐C chemokine receptor (CXCR6), affect tumor progression through different pathways, including leukocyte recruitment and function, cellular senescence, tumor cell proliferation, survival, invasion, and metastasis.^8^, ^9^, ^10^, ^11^, ^12^ It has been reported that CXCL16 is critical for evaluating the prognosis of several malignant tumors.^13^ CXCL16 is also recognized as a hypoxic stress marker in the tumor microenvironment based on its relationship with hypoxia‐induced factor.^14^ It has also been reported that hypoxic environments are closely associated with angiogenesis.^15^, ^16^, ^17^ Thus, these findings indicate the possibility of CXCL16 reflecting the concentration of VEGF and the efficacy of anti‐VEGF therapy. However, little is known about the effect of serum CXCL16 levels in lung cancer patients. In this article, serum CXCL16 levels in lung cancer patients and expression of CXCL16 in lung cancer samples were examined to evaluate the clinical implications for NSCLC patients.

## Methods

### Study design and patient samples

The expression of CXCL16 in tumor tissues and levels in sera were assessed. Sera were collected from patients with NSCLC at Yokohama City University from October 2010 to August 2016. The eligible patients were above 18 years and diagnosed histologically as having NSCLC. They were classified by multimodal approaches such as whole‐body computed tomography (CT), head magnetic resonance imaging (MRI), and/or fluorine‐18 fluorodeoxyglucose positron emission tomography (FDG‐PET), as being at an advanced stage of the disease or having postoperative recurrence (The International Association for the Study of Lung Cancer seventh edition of Tumor Node Metastasis Staging classification), and they were cytotoxic agent‐naïve. Sera from these patients were collected just before the initiation of chemotherapy, before the next cycle, and at the time of evaluation of the efficacy of chemotherapy by imaging. Serum samples were separated from peripheral venous blood and maintained at −80°C until analysis. Collected data included demographic characteristics at the time of the initiation of chemotherapy, disease stage at diagnosis, history of operation, the treatment regimen, the mutational status of epidermal growth factor receptor (EGFR)/echinoderm microtubule‐associated protein‐like 4 ‐ anaplastic lymphoma kinase (EML4‐ALK), progression‐free survival (PFS; time from administration to disease progression), and overall survival (OS; time from administration to death from any cause). Tumor responses were assessed according to Response Evaluation Criteria in Solid Tumors (RECIST) v1.1. Patients' characteristics are shown in Table 2.

Serum CXCL16 levels were measured as described below. The relationship between the starting value and change in the ratio of serum CXCL16 levels, therapeutic effects, PFS, and OS were analyzed. After the measurement of serum CXCL16 levels, patients with similar pretreatment serum CXCL16 levels and similar changes in serum CXCL16 levels post‐treatment were assigned into two groups. Data analysis was computed using GraphPad Prism version 6 for Mac (GraphPad Software, San Diego). The research was conducted per the 1964 Declaration of Helsinki and amendments. This study was approved by the institutional review board at Yokohama City University (Approved No. B140703019). All participants gave written informed consent before their inclusion in the study.

## ELISA

The value of serum CXCL16 level was measured using human CXCL16 ELISA kit (Human CXCL16 Quantikine ELISA Kit, R&D Systems, Minneapolis, MN, USA), following the manufacturer's protocol, and all analyses were performed in duplicates for the assessment of the interassay variations. Briefly, to 100 μL of assay diluent (provided with the kit), 50 μL of standards, controls, and serum samples were added in different wells of an ELISA plate and incubated for 2 hours at room temperature. This was followed by washing with quantikine wash buffer (provided with the kit) four times. Then, 200 μL of conjugated antibody was added to each well, and the plate was further incubated for 2 hours at room temperature, and the plate was washed thereafter as described previously. Next, 200 μL of substrate solution was added, and the plate was incubated for 30 minutes in the dark at room temperature. After incubation, 50 μL of stop solution (2N H_2_SO_4_) was added to each well, and the optical density was read using a Multi‐Detection Microplate Reader (Powerscan HT, DS Pharma Biomedical, Osaka, Japan) at 450 nm.

### Immunohistochemistry

Immunohistochemical analysis for the expression of CXCL16 and VEGF‐A was performed using lung cancer tissue microarray (US Biomax, Inc., Rockville, MD, USA). All tissue samples were collected following appropriate ethical standards, and in line with the Health Insurance Portability and Accountability Act approved protocol (USA). Written informed consent was obtained from all donors.

The tissue microarray blocks were fixed with formalin and cut into 5 μm thick sections and stained to check for the presence of CXCL16 (mouse monoclonal, GTX632502, dilution 1:100, GeneTex, San Antonio, TX) and VEGF‐A (rabbit monoclonal, ab27620, prediluted, Abcam, UK) following the manufacturer's protocol. Endogenous peroxidase activity was temporarily blocked after deparaffinization. Antigen retrieval was accomplished using citric acid buffer at pH 6.0 in a heat‐resistant container at 121°C for 10 minutes. To minimize nonspecific staining, slides were incubated with fetal goat serum at 37°C for 15 minutes. After blocking, slides were incubated with each of the primary antibodies against the antigens mentioned above at 37°C for 1 hour. After the second incubation with peroxidase linked antibody (rabbit polyclonal, 424 141, Nichirei Biosciences, Tokyo, Japan), substrate chromogen was added, and the specimens were lightly counterstained with hematoxylin. The expression levels of CXCL16 and VEGF‐A in cancer cells or stromal cells were statistically compared using chi‐square tests.

### Statistical analysis

Fisher's exact tests were conducted for the comparison of CXCL16 and VEGF expression in lung cancer tissue array and the comparison of the response rate between the low and high CXCL16 groups. Statistical analyses of the difference in serum CXCL16 levels between healthy people and NSCLC patients and the comparison of the pre‐ and post‐chemotherapy serum CXCL16 levels were conducted using a two‐sided student's *t*‐test. *P*‐values <0.05 were considered statistically significant. The Kaplan‐Meier method was used to analyze the PFS or OS among the high and low serum CXCL16 groups. Log‐rank tests were used to determine the statistical significance in survival curves.

## Results

### CXCL16 was expressed in stromal cells at the cancer site, whereas VEGF‐A was expressed in cancer cells

We assessed the expression of CXCL16 and VEGF‐A and conducted analysis using lung cancer tissue microarray (US Biomax, Inc., Rockville, MD, USA). Differences in localization between CXCL16 and VEGF‐A expression were clarified in the samples identified in lung cancer tissue array. From the results, 86 and 16 tissue samples were detected for the expression of CXCL16 and VEGF‐A, respectively. The expression of CXCL16 was dominant in the stromal cells, whereas VEGF‐A tended to be expressed more in the cancer cells (Figure SS1). Thus, these markers were significantly different in expression patterns (Table 1).

**Table 1:** CXCL16 and VEGF expression in lung cancer tissue array. Statistical analysis was done by Fisher's exact test

	Expression level	CXCL16 (n, %)	VEGF (n, %)	*P‐*value
The expression levels of CXCL16 and VEGF in cancer cells				
Cancer cells	High	1 (1)	4 (25)	0.002
	Low	36 (42)	7 (44)	
	None	49 (57)	5 (31)	
The expression levels of CXCL16 and VEGF in stromal cells				
Stromal cells	High	18 (21)	0 (0)	0.002
	Low	42 (49)	3 (19)	
	None	26 (30)	13 (81)	

CXCL16, chemokine (C‐X‐C motif); VEGF, vascular endothelial growth factor.

### Serum CXCL16 was significantly increased in patients with NSCLC compared to that in healthy volunteers

**Figure 1:**
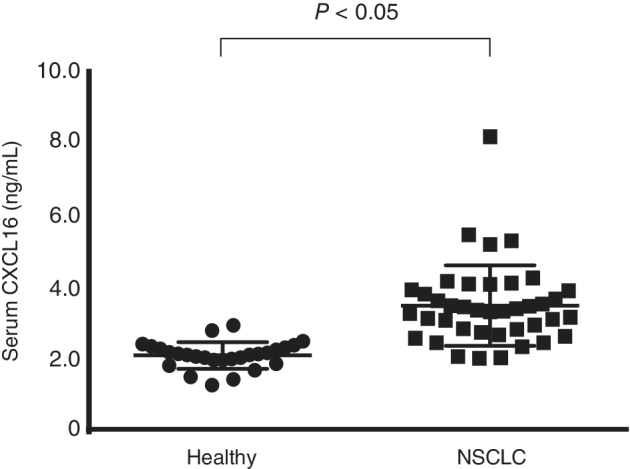
Comparison of serum CXCL16 between healthy volunteers and non‐small cell lung cancer patients (NSCLC). Comparison of serum CXCL16 concentration between healthy volunteers (*n* = 27) and NSCLC (*n* = 40). The lines indicate the median value for each group. The concentrations were statistically compared with the student *t*‐test. CXCL16, chemokine (C‐X‐C motif) ligand 16.

This study enrolled 40 patients with NSCLC. Patients' characteristics are summarized in Table 2. The clinical characteristics of the patients were as follows: the median age was 66.5 (range: 38–85) years, the male/female ratio was 21 (52.5%)/19 (47.5%), there were 34 adenocarcinomas (85.0%), and the majority of the clinical stages were stage IV with 34 cases (85.0%). Cytotoxic agents and tyrosine kinase inhibitors (TKI) were received by 27 and 13 patients, respectively. Of patients who received cytotoxic agent chemotherapy, 14 received treatment with bevacizumab, and 13 patients received treatment without bevacizumab. Serum CXCL16 levels in the 40 patients with NSCLC and 27 healthy volunteers were compared. Fig 1 shows the concentration of CXCL16 in serum detected by ELISA. The median serum CXCL16 level before treatment in patients with NSCLC was 3.5 (2.0–8.1) ng/mL, which was significantly higher than that in age‐matched healthy adults (2.1 ng/mL, *P* < 0.05).

**Table 2:** Patient characteristics

Patient demographics (*n* = 40)		
Age at diagnosis	Median (range)	66.5 (38–85)
Sex	Male	21 (52.5%)
	Female	19 (47.5%)
Smoking	Never	14 (35.0%)
	Ever	18 (45.0%)
	Current	8 (20.0%)
Stage	IIIB	3 (7.5%)
	IV	34 (85.0%)
	Postoperative recurrence	3 (7.5%)
Histology	Adenocarcinoma	34 (85.0%)
	Squamous cell carcinoma	4 (10.0%)
	NSCLC	1 (2.5%)
	LCNEC	1 (2.5%)
PS	0	14 (35.0%)
	1	12 (30.0%)
	2	5 (12.5%)
Regimen	Platinum doublets with BEV	13 (32.5%)
	Platinum doublets	14 (35.0%)
	EGFR‐TKI	11 (27.5%)
	ALK‐TKI	2 (5.0%)

ALK, anaplastic lymphoma kinase; BEV, bevacizumab; EGFR, epidermal growth factor receptor; LCNEC, large cell neuroendocrine carcinoma; NSCLC, non‐small cell lung cancer; PS, performance status; TKI, tyrosine kinase inhibitor.

### Serum CXCL16 was significantly decreased after successful treatment with bevacizumab

**Figure 2:**
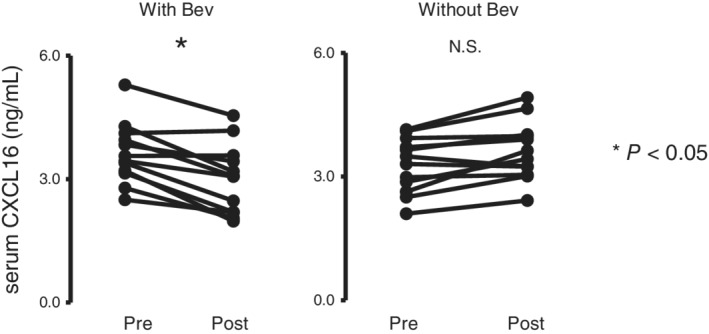
Changes in serum CXCL16 levels in chemotherapy‐sensitive patients between pre‐ and post‐treatment. (**a**) Serum CXCL16 levels at pre‐ and post‐chemotherapy, including bevacizumab among patients who achieved stable disease, partial response or complete response after 2–4 courses of treatment (*n* = 12). CXCL16 levels were significantly decreased. (**b**) Serum CXCL16 levels among those undergoing therapy without bevacizumab (*n* = 12). *P < 0.05 (compared with serum CXCL16 level before treatment). Bev, bevacizumab; Chemo, chemotherapy; CXCL16, chemokine (C‐X‐C motif) ligand 16.

Fig 2 shows the difference in serum CXCL16 values pre‐ and post‐chemotherapy when patients were treated with or without bevacizumab. Serum CXCL16 levels was significantly decreased in patients showing stable disease, partial response, or complete response after 2–4 courses of treatment with platinum‐doublet plus bevacizumab concurrently, whereas CXCL16 levels did not decrease in patients treated using platinum‐doublet without bevacizumab (Fig 2). These results indicate that serum CXCL16 levels may play an important role in the efficacy of anti‐VEGF therapy.

### CXCL16 may predict the efficacy of bevacizumab in addition to chemotherapy

**Figure 3:**
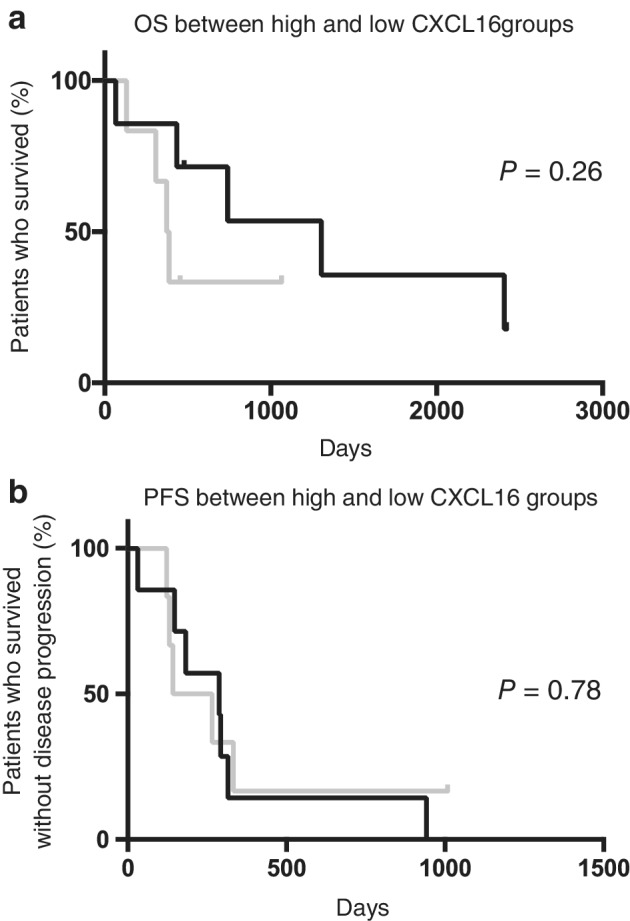
Overall survival and progression‐free survival after chemotherapy, including bevacizumab. (**a**) Comparison of overall survival (OS) after chemotherapy, including bevacizumab based on low or high CXCL16. (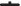) CXCL 16 low (*N* = 7) mOS 1306 (days). (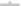) CXCL 16 high (*N* = 7) mOS 380 (days). (**b**) Comparison of progression‐free survival (PFS) after chemotherapy, including bevacizumab based on low or high CXCL16. (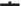) CXCL 16 low (*N* = 12) mPFS 288 (days). (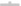) CXCL 16 high (*N* = 12) mPFS 204 (days). OS and PFS were statistically analyzed by the log‐rank test. CXCL16, chemokine (C‐X‐C motif) ligand 16; mOS, median overall survival; mPFS, median progression‐free survival; VEGF, vascular endothelial growth factor.

To assess the predictive value of serum CXCL16 levels in patients treated with anti‐VEGF therapy, patients who received bevacizumab in addition to chemotherapy were assigned into two groups according to their serum CXCL16 concentrations (cutoff value was 3.45 ng/mL, Fig 3). The response rate (RR) was significantly higher in the low CXCL16 group (RR = 85.7%) than in the high CXCL16 group (RR = 16.5%) (*P* = 0.029, Table 3). However, there were no significant differences in OS (Fig 3a) and PFS (Fig 3b).

**Table 3:** The difference of response after treatment between CXCL16 low and high group

	CXCL16 low	CXCL16 high	*P*‐value
CR	0	1	
PR	6	0	
SD	0	5	
PD	1	0	
RR	85.7%	16.7%	0.029
DCR	85.7%	100%	n/a

Statistical analysis was performed using the Fisher's exact test.

CR, complete response; CXCL16, chemokine (C‐X‐C motif); DCR, disease control rate; PD, progressive disease; PR, partial response; RR, response rate; SD, stable disease.

### Significant decrease in CXCL16 after chemotherapy is related to longer OS among NSCLC patients

**Figure 4:**
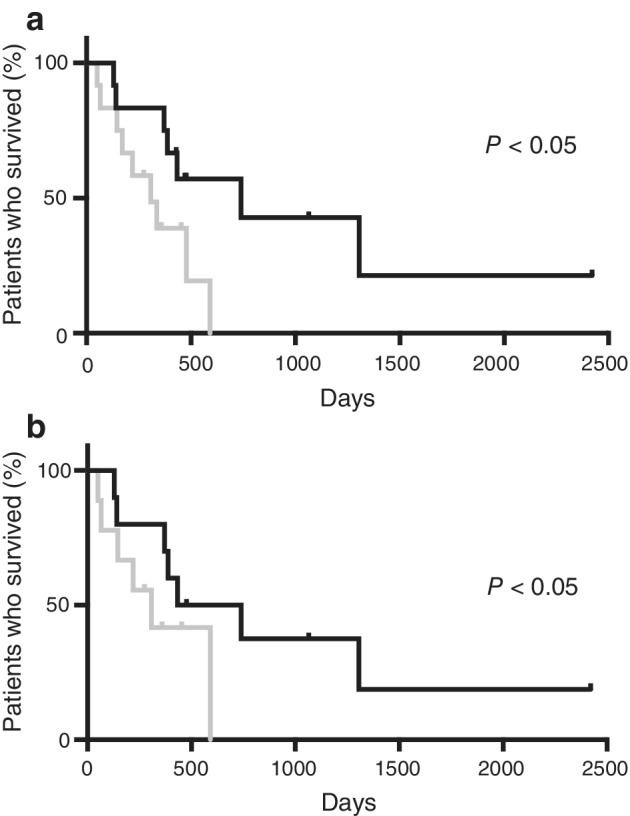
Overall survival based on the difference in CXCL16 after chemotherapy. Overall survival (OS) for patients with large difference in CXCL16 between pre‐ and post‐chemotherapy versus those with low difference. The cutoff value of the CXCL16 concentration was 0.07 ng/ml. Statistical comparison was done using the log‐rank test. CXCL16, chemokine (C‐X‐C motif) ligand 16. (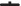) large decrease (*N* = 12) mOS 453 (days) (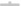) small decrease (*N* = 12) mOS 291 (days). (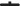) large decrease (*N* = 10) mOS 411 (days) (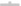) small decrease (*N* = 9) mOS 274 (days).

When OS in patients treated with chemotherapy was evaluated against the difference in CXCL16 levels pre‐ and post‐chemotherapy, it was observed that patients who showed a significant decrease in CXCL16 levels after chemotherapy experienced a longer OS than those who showed a small decrease (cutoff value was 0.07 ng/mL, Fig 4a). In the groups with large and small decrease in CXCL16 levels, nine and three patients were treated with cytotoxic agents and bevacizumab, respectively. Other patients were treated with cytotoxic agents. These findings were consistent with the analysis findings when squamous cell lung cancer patients were excluded (Fig 4b). Hence, pretreatment with serum CXCL16 and VEGF‐A concentrations had no significance as prognostic markers (data not shown); a larger decrease in serum CXCL16 levels post‐chemotherapy may imply a better prognosis. These data revealed the relationship between serum CXCL16 level and chemotherapy with bevacizumab.

## Discussion

VEGF is an important factor in cancer angiogenesis and is upregulated by oncogene expression, various growth factors, and hypoxia. VEGF targeted therapy, including bevacizumab, which is the humanized antibody for VEGF‐A, offers clinical benefits to patients with several types of cancer including colon cancer, breast cancer, and NSCLC. This is because it prevents angiogenesis in tumors thereby halting the development and growth of these cancers.^2^, ^17^, ^18^ CXCL16 is a chemokine that belongs to one of the CXC chemokine families and is usually produced by dendritic cells. In the tumor microenvironment, the CXCL16 and CXCR6 axes enhance tumor progression through the regulation of proangiogenic factor expression with the AKT/mTOR pathway in prostate cancer,^19^ the enhancement of precancerous inflammation in hepatocellular carcinoma,^20^ and the promotion of cell migration by hypoxia‐induced factor 1 alpha (HIF‐1α) in breast cancer.^21^ The mechanism of proliferation and migration of cancer cells suggests that CXCL16 induces angiogenesis in an autocrine manner via ERK, Akt, and p38 pathways, and VEGF secretion by HIF‐1α modulation.^22^ Our data revealed that serum CXCL16 levels increased among patients with NSCLC (Fig 1), and its location of expression was different from that of VEGF, which is more likely to be seen at the cancer site as previously reported (Table 3).^14^ Moreover, based on the results showing an association between decreases in CXCL16 levels and improvement of prognosis, serum CXCL16 level was identified as a potential biomarker for the chemotherapy efficacy, especially anti‐VEGF therapy (Fig 4).

It was reported that serum CXCL16 levels were elevated in patients with prostate cancer^23^ or pancreatic cancer^24^ than in those in healthy control patients. Recent data indicated that CXCL16 was also upregulated in the case of NSCLC (Fig 1). This is the first report evaluating the changes in serum CXCL16 levels pre‐ and post‐chemotherapy in patients with NSCLC. Interestingly, a larger difference in serum CXCL16 was related to longer survival. Several studies have been performed to detect predictive biomarkers for anti‐VEGF therapy efficacy, but at the moment, none have proven to have clinical benefits in the treatment of patients with advanced lung cancer. Our data highlights the possibility that serum CXCL16 levels can be monitored to estimate the treatment efficacy of anti‐VEGF therapy in the future.

The current study has several limitations. This was a single‐center based study with a small number of patients, who are only Japanese. Second, treatments were chosen by the principal physician. To overcome these limitations, multicenter international randomized trials should be conducted to evaluate the utility of serum CXCL16 levels as a biomarker to predict the efficacy of chemotherapy with the inclusion of anti‐VEGF treatment.

In conclusion, our data suggested that serum CXCL16 levels can be identified as a potential biomarker for the efficacy of chemotherapies, such as bevacizumab and other anti‐VEGF therapies for patients with advanced NSCLC.

## Disclosure

The authors declare no conflicts of interest.

## Supporting information


**Figure S1** Differences in the expression between VEGF‐A and CXCL16 in non‐small cell lung cancer patients by immunohistochemistry. Immunohistochemical analysis of the expression of VEGF‐A and CXCL16 was evaluated using lung cancer tissue microarray (US Biomax, Inc., Rockville, MD, USA). CXCL16 was expressed in both stromal and cancer cells, whereas VEGF tended to be expressed in cancer cells.
