# Supplementary figures and images for: The clinical significance of CXCL16 in the treatment of advanced non‐small cell lung cancer
Source: Thorac Cancer. 2020 Mar 12;11(5):1258–64. doi: 10.1111/1759-7714.13387 (PMC7180569; doi:10.1111/1759-7714.13387)

## Supplementary Figure 1

VEGF-A

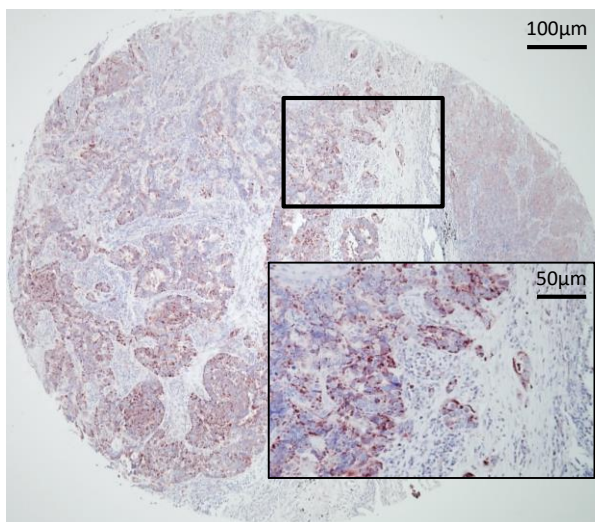

CXCL16

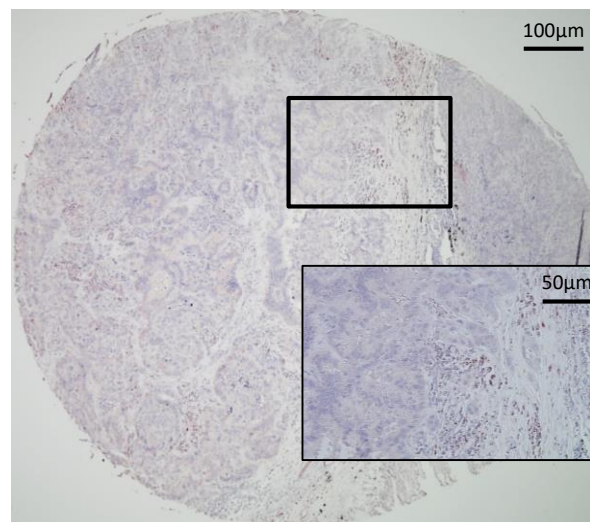

Supplement: Supplementary file 1 — Figure S1 Differences in the expression between VEGF‐A and CXCL16 in non‐small cell lung cancer patients by immunohistochemistry. Immunohistochemical analysis of the expression of VEGF‐A and CXCL16 was evaluated using lung cancer tissue microarray (US Biomax, Inc., Rockville, MD, USA). CXCL16 was expressed in both stromal and cancer cells, whereas VEGF tended to be expressed in cancer cells. [file TCA-11-1258-s001.pdf]
